# Persistence with tamoxifen and aromatase inhibitors in Germany: a retrospective cohort study with 284,383 patients

**DOI:** 10.1007/s00432-022-04376-5

**Published:** 2022-09-23

**Authors:** Niklas Gremke, Sebastian Griewing, Saket Chaudhari, Swati Upadhyaya, Ivan Nikolov, Karel Kostev, Matthias Kalder

**Affiliations:** 1 Department of Gynecology and Obstetrics, University Hospital Marburg, Philipps-University Marburg, Baldingerstraße, 35043 Marburg, Germany; 2 IQVIA, Bangalore, India; 3 Department of Gynecology, Herz Jesu Clinic, Fulda, Germany; 4 Epidemiology, IQVIA, Frankfurt, Germany

**Keywords:** Breast cancer, Persistence, Endocrine therapy, Tamoxifen, Aromatase inhibitors, Germany

## Abstract

**Purpose:**

The aim of this study was to analyze the persistence of women on tamoxifen (TAM) and aromatase inhibitors (AIs) in Germany, and to investigate possible determinants of non-persistence.

**Methods:**

The present retrospective cohort study was based on the IQVIA longitudinal prescription database (LRx). The study included women with an initial prescription of TAM or AIs (anastrozole, letrozole, and exemestane) between January 2016 and December 2020 (index date). Kaplan–Meier analyses were performed to show the persistence for TAM and AI, using a therapy gap of 90 or 180 days, respectively. A multivariable Cox proportional hazards regression model was further used to estimate the relationship between non-persistence and drug prescription (AI versus TAM), age, and the specialty of the physician initiating therapy (gynecologist, oncologist, or general practitioner).

**Results:**

Up to 5 years after the index date, only 35.1% of AI and 32.5% of TAM patients were continuing therapy when therapy discontinuation was defined as at least 90 days without therapy. Using a 180-day therapy gap, 51.9% of AI and 50.4% of TAM patients remained on therapy after 5 years. Cox regression models reveal that initial therapy with TAM (HR 1.06, 95% CI 1.04–1.07), therapy initiation by oncologists (HR 1.09, 95% CI 1.07–1.11), or general practitioners (HR 1.24, 95% CI 1.21–1.27) and age ≤ 50 (HR 1.08, 95% CI 1.06–1.10) were significantly associated with an increased risk of therapy discontinuation.

**Conclusion:**

Overall, the present study indicates that persistence rates are low in all age groups for both TAM and AI treatment. We found several factors (e.g., physician specialty, younger age, and type of endocrine therapy) to be associated with an increased risk for non-persistence.

## Introduction

Breast cancer (BC) is the most common cancer among women in Germany, with about 69,000 new cases annually; 18,591 women died of BC in 2018 (Barnes et al. 2016). About 70% of all newly diagnosed BC are hormone receptor positive (HR+), for which the primary adjuvant treatment is endocrine therapy (ET) with either tamoxifen (TAM) or third-generation aromatase inhibitors (AIs), including letrozole, anastrozole, and exemestane (Franzoi et al. 2021). The mechanism of TAM involves acting as a selective estrogen receptor modulator (SERM), while AIs impede the conversion of androgens to estrogens, leading to estrogen deprivation and thus counteracting estrogen-promoted tumor growth (Johnston and Dowsett 2003). The standard ET with either TAM or AIs is taken daily for at least 5 years according to menopausal status, significantly reduces BC recurrence, and improves patient’s overall survival (Early Breast Cancer Trialists’ Collaborative 2015; Early Breast Cancer Trialists’ Collaborative et al. 2011; Font et al. 2022; Waks and Winer 2019). In the past, TAM was recommended as the first-line treatment in both pre- and postmenopausal women. However, based on encouraging results of several studies, AIs are replacing TAM as the initial adjuvant endocrine therapy in postmenopausal women and have also been approved in premenopausal women in combination with ovarian suppression for high-risk BC patients (Francis et al. 2018; Howell et al. 2005; Pagani et al. 2014). Despite their proven efficacies for preventing BC recurrence by 40%, about 50% of women take less than 80% of the prescribed dosage and up to 50% of BC patients discontinue their ET (Hadji et al. 2013; Moon et al. 2019; Peddie et al. 2021).

It is of note that the benefits obtained with adjuvant ET come at a cost. For example, the use of TAM is accompanied by several important toxicities, such as hot flashes, an increased risk of endometrial cancer, and thromboembolic events, whereas higher rates of arthralgias, myalgias, and osteoporosis-related bone fractures were observed for AIs (Early Breast Cancer Trialists' Collaborative 2015; Early Breast Cancer Trialists’ Collaborative et al. 2011; Franzoi et al. 2021; Group et al. 2009). Therefore, the occurrence of TAM and AI-related side effects can negatively affect a patient’s persistence with and adherence to ET, with persistence being defined as continuing to take medications for the prescribed time period (from initiation to discontinuation) and adherence as the extent to which a patient acts in accordance with the prescribed interval and dose of a dosing regimen (Cramer et al. 2008; Hadji et al. 2013). Recently, adherence to and persistence with ET in women with HR + BC have been intensively discussed in the literature, since non-adherence to ET has been shown to be significantly associated with an increased risk of disease recurrence (Font et al. 2019; Seneviratne et al. 2015), distant metastasis (Blanchette et al. 2020; Lee et al. 2019), and mortality (Inotai et al. 2021; Lao et al. 2019; Murphy et al. 2015). However, only a small number of studies published some time ago have analyzed the influence of age on persistence with TAM or AIs in Germany, and factors related to treatment discontinuation remain unclear (Jacob et al. 2016). Therefore, the aim of our study was to analyze persistence with TAM and all three available AIs using current data from the longitudinal LRx database and to investigate possible non-persistence factors among 284,383 German patients.

## Methods

### Database

The present retrospective cohort study was based on the IQVIA longitudinal prescription database LRx (Richter et al. 2015). This database comprises approximately 80% of prescriptions reimbursed by statutory health insurance funds in Germany. Data are available at the patient level, including information on the age and sex of the patients. All patient information is fully anonymized by the data provider in accordance with data privacy laws. Each available prescription includes full product information (e.g., brand, substance, package size, and product form) and dates dispensed. The database does not contain diagnoses or laboratory tests (Richter et al. 2015). Finally, this database has been used effectively in previous studies on persistence (Eisen et al. 2020; van den Boom and Kostev 2022).

### Study population and outcomes

This retrospective cohort study included women with an initial prescription of TAM or AIs (anastrozole, letrozole, and exemestane) between January 2016 and December 2020 (index date). No therapy switchers but only new patients were included. The study outcome measure was the rate of persistence within 5 years of the index date. Each patient was followed for up to 60 months (standard duration for ET) from the index date until therapy with TAM and AI ended or was discontinued. Therapy discontinuation was defined as at least 90 days without therapy. When patients switched from TAM to AI or vice versa within 180 days after the discontinuation of each therapy, they were further considered persistent. A sensitivity analysis was also performed using a 180-day therapy gap. The expected duration for each prescription was calculated on the basis of the package size, number of packages, and defined daily dose (DDD).

### Statistical analyses

Kaplan–Meier analyses were performed to show the persistence for TAM and AI separately for 90- and 180-day therapy gap. We further used a multivariable Cox proportional hazards regression model to estimate the relationship between initial drug (AI versus TAM), age, and the specialty of the physician initiating therapy (gynecologist, oncologist) and non-persistence. *P* values < 0.001 were considered statistically significant. Analyses were carried out using SAS version 9.4 (SAS institute, Cary, NC, USA).

## Results

### Basic characteristics of the study sample

This study included 284,383 patients. Of those, 156,006 received AI and 128,377 received TAM as an initial endocrine therapy. Baseline characteristics of the study population are shown in Table 1. Mean age was 69.0 (SD 12.3) in AI and 59.1 (SD 14.4) in TAM patients. The majority of patients were treated by gynecologists (68.6% of AI and 76.5% of TAM patients), while oncologists treated 11.1% of AI and 6.3% of TAM patients, and general practitioners were therapy initiators for 7.1% of AI and 6.6% of TAM patients.

**Table 1:** Basic characteristics of the study sample

Variable	Proportion among patients treated with aromatase inhibitors (%)	Proportion among patients treated with tamoxifen (%)
*N*	156,006	128,377
Age (mean, SD)	69.0 (12.3)	59.1 (14.4)
Age ≤ 50	8.607 (5.5)	39,023 (30.4)
Age 51**–**60	30,583 (19.6)	35,395 (27.6)
Age 61**–**70	44,023 (28.2)	24,274 (18.9)
Age > 70	72,793 (46.7)	29,685 (23.1)
Physician who initiated therapy		
Gynecologist	106,970 (68.6)	98,148 (76.5)
Oncologist	17,360 (11.1)	8057 (6.3)
General practitioner	11,012 (7.1)	8421 (6.6)
Other or unknown	20.664 (13.3)	13.751 (10.7)

Proportions of patients given in %, unless otherwise indicated

*SD* standard deviation

### Persistence analysis

**Fig. 1:**
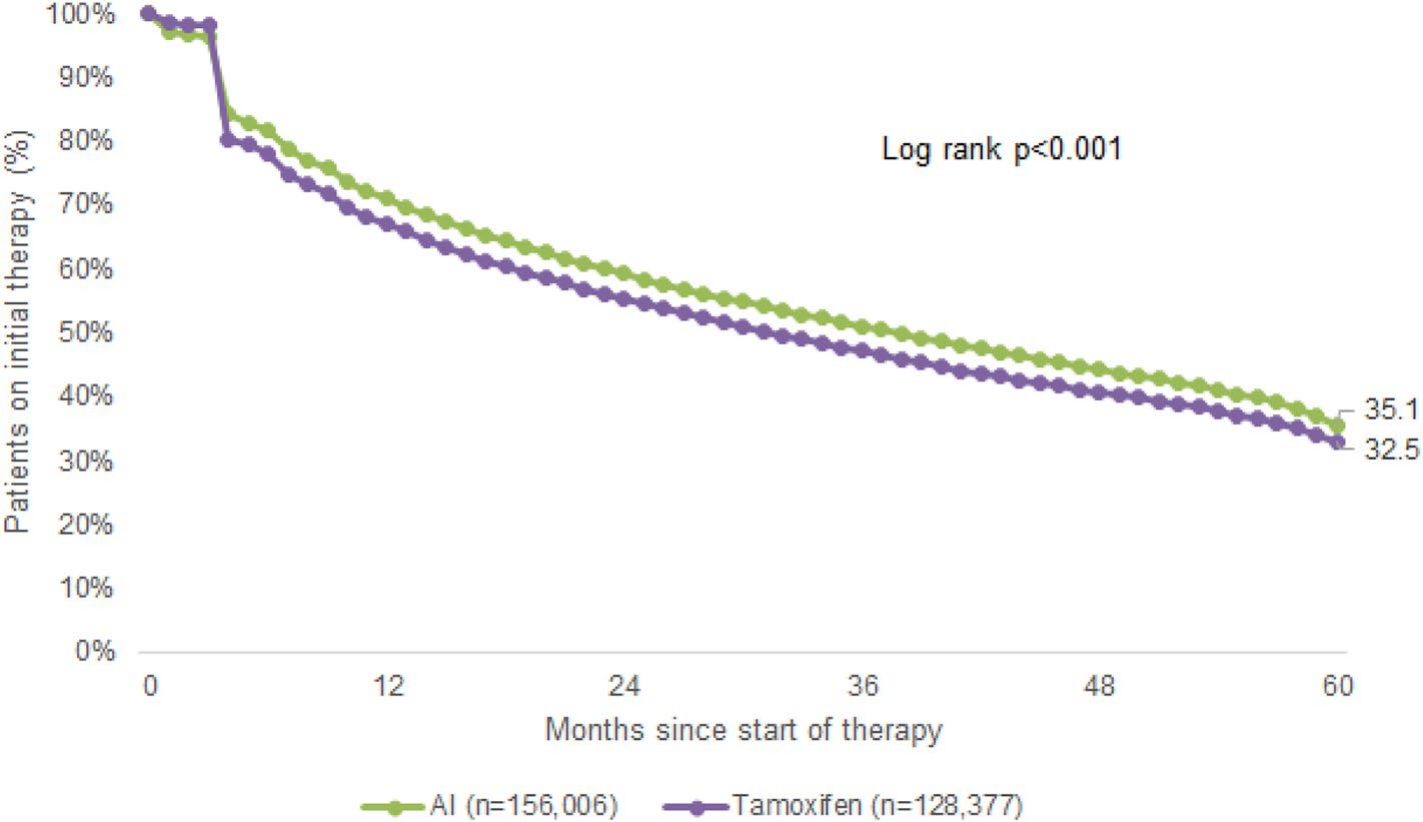
Kaplan–Meier curves for persistence in patients treated with tamoxifen and aromatase inhibitors (considered gap for therapy discontinuation = 90 days)

**Fig. 2:**
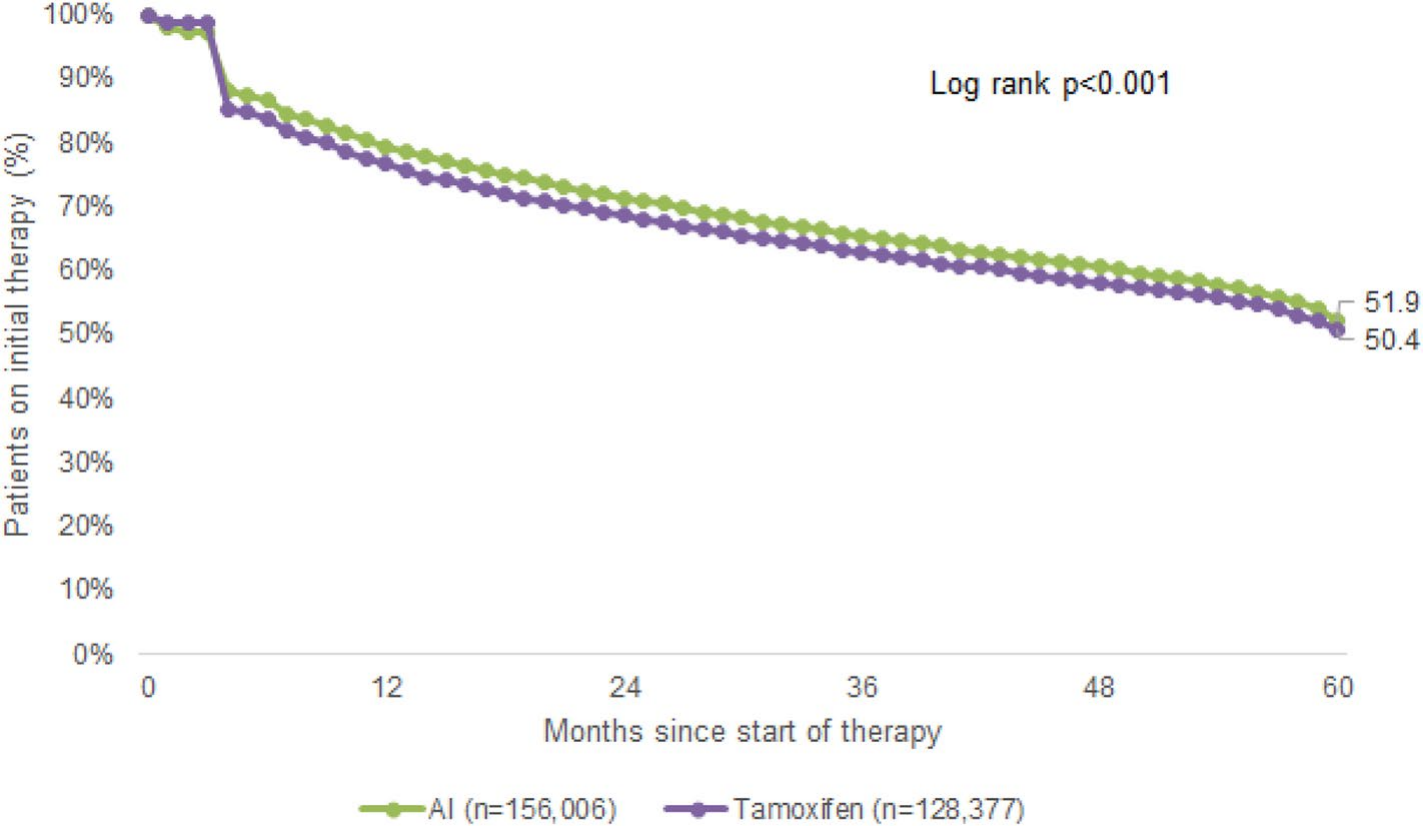
Kaplan–Meier curves for persistence in patients treated with tamoxifen and aromatase inhibitors (considered gap for therapy discontinuation = 180 days)

Up to 5 years after the index date, only 35.1% of AI and 32.5% of TAM patients were continuing therapy when therapy discontinuation was defined as at least 90 days without therapy (Fig. 1). In sensitivity analyses with a 180-day therapy gap, 51.9% of AI and 50.4% of TAM patients remained on therapy after 5 years (Fig. 2). During the therapy course, 8.8% of AI patients switched to TAM therapy within allowed gap of 90 days; 24.1% of TAM patients switched to AI.

The results of the Cox regression models are shown in Table 2. Initial therapy with TAM was associated with a slightly higher risk of therapy discontinuation (HR 1.06, 95% CI 1.04–1.07). Compared to the age group > 70, patients aged ≤ 50 were at slightly higher therapy discontinuation risk (HR 1.08, 95% CI 1.06–1.10), since age groups 51–60 and 61–70 were associated with a slightly lower discontinuation risk (HR 0.92, 95% CI 0.91–0.94) for 61–70 years and (HR 0.89, 95% CI 0.88–0.91) for 51–60 years. Finally, compared to gynecology, therapy initiated by oncologists (HR 1.09, 95% CI 1.07–1.11) and general practitioners (HR 1.24, 95% CI 1.21–1.27) was significantly associated with an increased risk of therapy discontinuation.

**Table 2:** Association between predefined variables and risk of therapy discontinuation (Cox regression models)

Variable	HR (95% CI)^a^	*P* value
Aromatase inhibitors	Reference	
Tamoxifen	1.06 (1.04–1.07)	< 0.001
Age ≤ 50	1.08 (1.06–1.10)	< 0.001
Age 51**–**60	0.92 (0.91–0.94)	< 0.001
Age 61**–**70	0.89 (0.88–0.91)	< 0.001
Age > 70	Reference	
Gynecologist	Reference	
Oncologist	1.09 (1.07–1.11)	< 0.001
General practitioner	1.24 (1.21–1.27)	< 0.001

^a^Multivariable Cox regression adjusted for age, physician specialty, and therapy

## Discussion

### Treatment discontinuation rates

In the present study, we analyzed persistence with ET in patients receiving either TAM or AI treatment up to 5 years after the index date, and found that only 32.5% of TAM and 35.1% of AI patients were still on therapy by the end of the fifth year of treatment where therapy discontinuation was defined as at least 90 days without therapy. When extending the therapy gap to 180 days, 50.4% of TAM and 51.9% of AI patients remained on therapy after the same treatment interval of 5 years. These results are in line with the current literature, in which discontinuation of treatment among patients treated with TAM ranged from 15 to 20% within the first few years of therapy and up to 31–60% by the end of year 5 (Owusu et al. 2008; van Herk-Sukel et al. 2010). In studies that analyzed both TAM and AI treatment, discontinuation rates ranged from 32 to 73% after 5 years of treatment (Guth et al. 2008, 2011; Murphy et al. 2012). However, it should be mentioned that we measured the highest possible persistence rate, since patients’ actual medication intake could not be controlled for this study, and it is already known that approximately 50% of patients do not take medications as prescribed (Brown and Bussell 2011). Furthermore, varying definitions of the therapy gap (between 45 and 180 days) and of persistence itself make it difficult to compare studies on ET persistence (Peddie et al. 2021).

In addition, Kaplan–Meier curves reveal a sharp drop in persistence within the first month of ET initiation and the current literature provides evidence that this early treatment discontinuation is mainly due to the occurrence of ET-related side effects impacting patients’ quality of life. In particular, it has been shown that early discontinuation of ET was associated with newly treated hot flashes (HR 2.1, 95% CI 1.3–3.3) whereby early discontinuation of ET was also accompanied with a dramatic increase in BC recurrence compared to those women who completed the recommended treatment course (Collin et al. 2021; Kemp et al. 2014). Furthermore, we observed that 51.9% of AI and 50.4% of TAM patients were persistent when therapy discontinuation was defined as at least 180 days without therapy. When a 90-day therapy gap was applied, 35.1% of AI and 32.5% of TAM patients remained on therapy after 5 years. Interestingly, Nekhlyudov et al. showed that longer gaps in treatment were associated with a lower probability of resuming ET. It is conceivable that some women stop ET temporarily and then resume therapy at a later point. However, the authors also reasoned that side effects or therapy costs may contribute to shorter interruptions in ET, while other patient, physician, and health system factors may contribute more to longer interruptions (Nekhlyudov et al. 2011).

### Factors associated with non-persistence

To address the issue that BC patients are at a significant risk of cancer recurrence even multiple years after initial diagnosis, several studies have analyzed the outcome of prolonged treatment with TAM or AIs for up to 10 years compared to the initial 5-year interval. In particular, continuing TAM treatment resulted in reduced rates of BC recurrence and mortality, but was also accompanied by increased rates of endometrial cancer and thromboembolic events (Davies et al. 2013). Extending AI treatment to 10 years resulted in significantly higher rates of BC disease-free survival, but no impact on overall survival was detected. Furthermore, as expected, toxic bone-related effects such as fractures and new-onset osteoporosis occurred more frequently among patients receiving AI for 10 years (Goss et al. 2016; Waks and Winer 2019). However, a problem with patients’ persistence rates emerged when ET was further extended to 10 years, since ET-related side effects are still known to negatively influence persistence with ET. In view of this, there is an urgent need to identify more (possibly modifying) factors that influence ET persistence to further improve patients survival benefits (Kahn et al. 2007; Lambert et al. 2018). In particular, we can show that the initiation of ET by oncologists (HR 1.09, 95% CI 1.07–1.11) and general practitioners (HR 1.24, 95% CI 1.21–1.27) was significantly associated with an increased risk of therapy discontinuation. In Germany, most BC patients are treated by gynecologists rather than oncologists or general practitioners, with gynecologists receiving special training and regular updates on the treatment of BC patients (Hadji et al. 2013). As it is conceivable that oncologists may treat metastasized and elderly BC patients with more severe comorbidities more frequently, the increased therapy discontinuation rate may be explained by more frequent side effects of ET and older age (Murphy et al. 2012). Moreover, chemotherapy application, which can reflect a more advanced BC stage, is normally administered by oncologists and was also associated with higher rates of non-persistence in several studies (Brito et al. 2014; Lambert-Côté et al. 2020; Schmidt et al. 2014).

We also found that women under the age of 50 had a slightly higher discontinuation risk (HR 1.08, 95% CI 1.06–1.10) than women over 70. These results are in line with those of previous studies, which found that young and elderly women were most likely to discontinue ET. For example, Hershman et al. reported in a large population-based study that women under 40 years old had the highest risk of discontinuation (HR 1.51; 95% CI 1.23–1.85). He et al. also found that BC patients at extremes of age (< 40 years; HR 1.39; 95% CI 1.08–1.78 and ≥ 65 years; HR 1.15; 95% CI 1.03–1.28) had the highest risk of discontinuation. There are a plethora of factors relating to fertility, the occurrence of more serious side effects upon ET initiation, body image concerns, etc. that may make young women with BC a vulnerable group with greater risk of recurrence upon therapy discontinuation (He et al. 2015; Hershman et al. 2010; Llarena et al. 2015; Sella and Chodick 2020).

Finally, we were able to show that initial therapy with TAM was associated with a slightly higher risk of therapy discontinuation (HR 1.06, 95% CI 1.04–1.07). There are several hypotheses that may explain this result: TAM is not as effective as AI in reducing BC patients’ recurrence risk (10-year BC recurrence risk of 22.7% for TAM vs 19.1% for AI) (Early Breast Cancer Trialists' Collaborative 2015). Once it has occurred under TAM treatment, BC recurrence is usually more aggressive and TAM-resistant than the primary tumor, and discontinuation may thus also be due to increased mortality (due to study limitations, death is also considered a therapy discontinuation) (Font et al. 2022; Yan et al. 2020). It is important to note that if patients switched ET from TAM to AI (or vice versa) within 180 days after the discontinuation of each therapy, they continued to be considered persistent. Therefore, a planned switch from one ET to another after completion of the initial regimen (e.g., 2–3 years of TAM) does not influence persistence rates as measured in our study. In particular, we show that 24.1% of TAM patients switched to AI, while only 8.8% of AI patients switched to TAM. These results are also in line with those of a prospective cohort study, conducted by Kwan et al. in which the authors analyzed ET class switching from AI to TAM (and vice versa) and found that of 2122 BC patients who started on AI therapy, 290 (13.7%) switched to TAM, and of 1143 patients who started on TAM therapy, 446 women (39.0%) switched to AI (Kwan et al. 2017).

In conclusion, persistence with all endocrine treatments in women was low and needs to be increased significantly. Further research is required to understand factors that influence persistence rate to improve patients’ outcomes in clinical practice.

#### Strengths and limitations

The present study is subject to several limitations that need to be mentioned. First, the LRx prescription database does not contain information about diagnoses and TNM status, so stratification by cancer stage and analysis of co-diagnoses was not possible. Second, no mortality data or information regarding the occurrence of side effects were available to evaluate the reasons for loss to follow-up. As a result, loss to follow-up may have been due to death, change of insurance fund, or change of residence, and not just discontinuation. Nevertheless, these potential limitations are offset by the strengths of our study, including the large number of patients, the long observation period, and the nationally representative data on drug prescriptions.

## Data Availability

Anonymized raw data are available upon reasonable request.
